# An automatic method to calculate heart rate from zebrafish larval cardiac videos

**DOI:** 10.1186/s12859-018-2166-6

**Published:** 2018-05-09

**Authors:** Chia-Pin Kang, Hung-Chi Tu, Tzu-Fun Fu, Jhe-Ming Wu, Po-Hsun Chu, Darby Tien-Hao Chang

**Affiliations:** 1 Department of Electronic Engineering, National Cheng Kung University, Tainan, 70101 Taiwan; 2 The Institute of Basic Medical Sciences, National Cheng Kung University, Tainan, 70101 Taiwan

**Keywords:** Zebrafish, Image processing, Heart rate

## Abstract

**Background:**

Zebrafish is a widely used model organism for studying heart development and cardiac-related pathogenesis. With the ability of surviving without a functional circulation at larval stages, strong genetic similarity between zebrafish and mammals, prolific reproduction and optically transparent embryos, zebrafish is powerful in modeling mammalian cardiac physiology and pathology as well as in large-scale high throughput screening. However, an economical and convenient tool for rapid evaluation of fish cardiac function is still in need. There have been several image analysis methods to assess cardiac functions in zebrafish embryos/larvae, but they are still improvable to reduce manual intervention in the entire process. This work developed a fully automatic method to calculate heart rate, an important parameter to analyze cardiac function, from videos. It contains several filters to identify the heart region, to reduce video noise and to calculate heart rates.

**Results:**

The proposed method was evaluated with 32 zebrafish larval cardiac videos that were recording at three-day post-fertilization. The heart rate measured by the proposed method was comparable to that determined by manual counting. The experimental results show that the proposed method does not lose accuracy while largely reducing the labor cost and uncertainty of manual counting.

**Conclusions:**

With the proposed method, researchers do not have to manually select a region of interest before analyzing videos. Moreover, filters designed to reduce video noise can alleviate background fluctuations during the video recording stage (e.g. shifting), which makes recorders generate usable videos easily and therefore reduce manual efforts while recording.

**Electronic supplementary material:**

The online version of this article (10.1186/s12859-018-2166-6) contains supplementary material, which is available to authorized users.

## Background

Congenital heart disease (CHD) is the most common congenital defect, which accounts for 3% of infant deaths [1]. To better prevent and treat this defect, a further understanding on the cardiac development is pre-requisite. Many automatic or semi-automatic analysis systems based on optical recording have been proposed for this purpose. Zebrafish is a vertebrate model with high genetic similarity to mammals [2]. The externally fertilized transparent embryos and larvae enable a real-time observation of cardiac development during embryogenesis. Embryos receive oxygen directly from the surrounding environment by passive diffusion before reaching seven-day post-fertilization (dpf). This characteristic allows for investigation on cardiac structure and function even when severe congenital heart defects are induced [3]. The aforementioned advantages make zebrafish an ideal model for studying cardiac development [4].

To date, there have been many methods that assess cardiac functions in zebrafish embryos/larvae [5–13]. These methods need manually pre-selected regions of interest (ROI) for satisfying results. To solve this problem, several digital image analysis methods have been developed to standardize and automate cardiac assessment in zebrafish embryos/larvae [14–20]. The aforementioned semi-automatic and automatic methods have been developed for videos obtained under different conditions. For example, the work of Luca et al. [15] used videos from resonant laser scanning confocal microscope, which is rather expensive for some laboratories. The issue of anesthetization has been addressed in Pylatiuk et al.’s work [16], but a recent work by Puybareau et al. [19] still chose to anesthetize the fish embryos with Tricaine. The contribution of these computational methods is to accurately estimate heart rate for a practical acquisition process.

Although the videos were obtained under different conditions, all of the aforementioned methods have to face two important issues after video acquisition: (a) identification of the heart area and (b) image processing to enhance heartbeat signals in the video. This work has developed a novel method for heart rate estimation. For the first issue, this work proposes a half-heart mask to identify the ROI. For the second issue, this work proposes that Empirical Mode Decomposition (EMD) could be used as a high-pass filter to reduce video noise. Another contribution of this work is the comparison strategy between the proposed method and manual counting. This work designed an experiment to quantify manual error, which was used as a reference to evaluate the computational error. The experimental results show that the proposed method achieves the same error level as human.

This article is organized as follows. Section 2 describes the proposed method, which includes three major steps. Section 3 describes its performance, including the details of zebrafish larva preparation, video acquisition and an experiment to compare the calculated heartbeat rate with manual counting. Section 4 concludes this work and discusses possible extensions.

## Methods

**Fig. 1:**
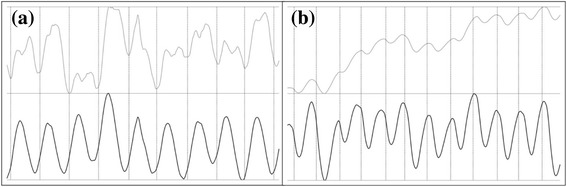
Heartbeat signals (h-signals): (**a**) a zebrafish larva video (ID: MVI_5280) in which each heartbeat corresponds to two brightness intensity peaks; (**b**) a zebrafish larva video (ID: MVI_5269) of which the zebrafish larva slid. The x-axis is frame index; the y-axis is brightness intensity of that frame. Light (the top half) and dark (the bottom half) curves indicate brightness intensities before and after, respectively, the proposed processing steps. Vertical dotted lines indicate the counting timings of the operator when he recognized a heartbeat through the video

**Fig. 2:**
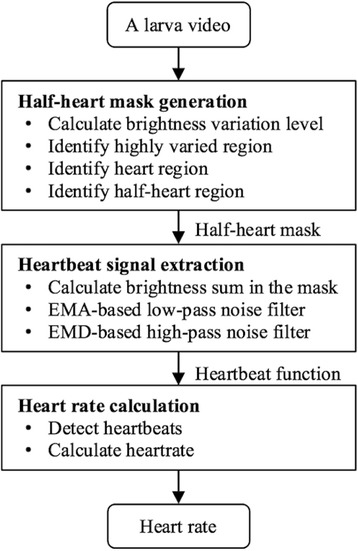
Flow chart of the proposed heart rate calculation

The concept of the proposed heart rate calculation is based on brightness intensity of each video frame. Fig. 1 shows that the brightness intensity through a video has a repetitive pattern related to heartbeats. This brightness intensity function is denoted heartbeat signal (h-signal) in this article. Fig. 2 shows the workflow of the proposed method. For a zebrafish larva video, the first step is to generate a mask where the brightness intensity is calculated. The second step is to extract an h-signal. The third step is to calculate a heart rate from the extracted h-signal.

### Half-heart mask generation

**Fig. 3:**
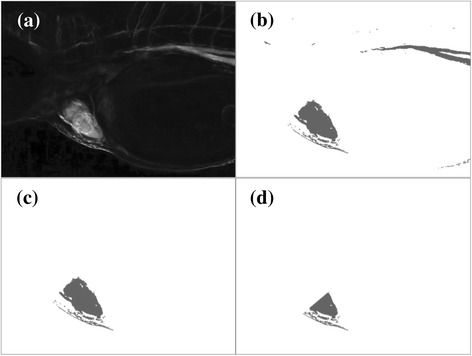
Outputs of the four filters in the proposed half-heart mask generation step: (**a**) variation level of brightness intensity (brighter pixels have higher variation); (**b**) highly varied region; (**c**) heart mask; (**d**) half-heart mask. The outputs require the information of all frames of a video and are generated only once for each video

Figure 3a shows the variation level of brightness intensities of all frames in a video. Extracting the h-signal from the entire video region includes brightness intensity from uninteresting regions, such as the bright pixels at the top right of Fig. 3a. They seriously affect the extracted h-signal but are weakly related to the heartbeats. A common practice to solve this problem is pre-selecting the heart region as a mask to screen out uninteresting regions. However, this practice requires manual intervention, which is not applicable for an automatic pipeline.

This work developed a procedure for half-heart mask generation, which consists of four filters. Given a zebrafish larva video, the first filter is to calculate, for each pixel, the variation level of brightness intensity through the video. This filter first converts each video frame to a grayscale image. In this work, 12 color-to-grayscale methods analyzed in [21] have been tested. The Luma method [22] without Gamma correction yielded the best performance and was adopted in this work. The performance of using the 12 color-to-grayscale methods can be found in the Additional file 1. After this conversion, each pixel in the frame has a brightness intensity, *y*. This work defines the variation level of brightness intensity of a pixel at (*i*, *j*) in an *n*-frame video as the variation of the *n*-1 brightness intensity differences of two consecutive frames at that pixel:

$$ \frac{1}{n-1}\sum \limits_{k=1}^{n-1}\left|{y}_{i,j,k+1}-{y}_{i,j,k}\right| $$

where *y*_*i,j,k*_ is the brightness intensity of pixel (*i*, *j*) in the *k*-th video frame. The variation levels of all pixels for a video are normalized to [0,255] so that they can be rendered to a grayscale image as shown in Fig. 3a, which is the output of the first filter.

The second filter is to find a region in which the brightness intensity varies drastically. This filter converts the grayscale image of variation levels into a binary mask by setting a percentage threshold, *θ*. The *θ*% pixels with the highest variation levels are converted to black (region of interest), otherwise are converted to white (region of non-interest). Since the target area of interest is heart, the ideal *θ* of a zebrafish larva video depends on its heart-to-video ratio: the area ratio of the heart region to the entire video. The heart-to-video ratios of the 32 testing videos collected in this work varied from 0.9% to 10.0%. This work used *θ* = 4 for all testing videos. The result of this filter is a binary mask as shown in Fig. 3b.

**Fig. 4:**
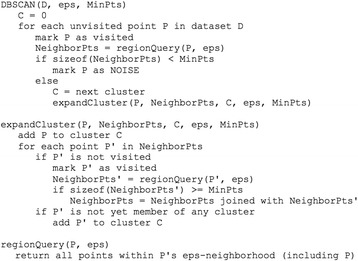
Pseudocode of the DBSCAN algorithm

The third filter is to further reduce the archipelago-like mask of Fig. 3b to focus on the heart region. The concept of this filter is to retrieve the biggest circle-like island. Long and narrow islands, even with large area, are not the region of interest. Invoking region-growing algorithms can solve this problem [23, 24]. The DBSCAN algorithm [25], which clusters pixels by “pixel density”, was adopted for this task. The nature of DBSCAN inhibits the growth of long and narrow clusters, because their pixel densities are difficult to maintain during the scanning process. Another benefit of DBSCAN is that the number of clusters is not pre-required. By applying neighborhood expansion, DBSCAN can filter out noise (i.e. spots in an image) to accurately recognize clusters of arbitrary size [26]. The DBSCAN algorithm defines core pixels based on two parameters, epsilon (*eps*) and minimum number of points (*minPts*). For a black pixel, *q,* its *eps*-neighborhood contains pixels of which the distance to *q* is less than *eps*. If the *eps*-neighborhood has more than *minPts* pixels, *q* is defined as a core pixel. The scanning process of DBSCAN picks an arbitrary pixel *p* from the un-scanned black pixels to start. If *p* is a core pixel, *p* and its *eps*-neighborhood form a new island. All pixels in this island are then scanned and their *eps*-neighborhoods are added into the island. The newly added pixels are also scanned to see if more pixels can be added to the island. This scanning process is conducted recursively until no more pixels can be added to island. If *p* is not a core pixel, *p* is considered as a noise and assigned to no island. Then, DBSCAN moves to the next un-scanned black pixel and stops when all black pixels have been scanned. Figure 4 shows the pseudocode of DBSCAN. This work used *eps* = 20 and *minPts* = 300 for all testing videos, of which the video resolution was 1280 X 720. If the video resolution changes, *minPts* should be adjusted because the number of pixels in the same area changes. The *eps* should also be adjusted to maintain the pixel density (*minPts*/*eps*^*2*^). For example, if a video resolution changes from 1280 X 720 to 640 X 360 (i.e. area becomes 1/4), the *minPts* is suggested to be 300/4 = 75 and the *eps* is suggested to be 20/sqrt(4) = 10. The result of this filter is the largest island of DBSCAN as shown in Fig. 3c, which is close to the heart.

The fourth filter is to split the heart mask of Fig. 3c into halves, corresponding to the atrial and ventricular of a zebrafish heart. When most of the blood cells stay in atrial, the brightness intensity in the atrial region reaches a minimum but that in the ventricular region reaches a maximum. Therefore, the brightness intensity in the entire heart region falls into two states, where a heartbeat might correspond to two brightness intensity peaks as the light curve in Fig. 1a. To solve such a double-peak issue, this work aims to extract the brightness intensity from either the atrial or ventricular rather than from the entire heart. To split the heart mask into two halves, this filter first calculates the brightness intensity centroid in the heart mask of each video frame. An *n*-frame video results in *n* centroids. The *k*-means algorithm [27] is then invoked to cluster the *n* centroids into the two clusters: one contains frames where most blood cells are in the atrial and the other contains frames where most blood cells are in the ventricular. The *k*-means algorithm partitions centroids based on their locations into *k* disjoint clusters. When clustering *n* data points into *k* cluster, the *k*-means algorithm minimizes the following equation:

$$ \sum \limits_{i=1}^k\sum \limits_{j=1}^n{w}_{i,j}\cdotp {\left\Vert {c}_i-{p}_j\right\Vert}^2 $$

where *c*_*j*_ is the center of the *i*-th cluster, *p*_*j*_ is the *j*-th data point, and *w*_*i,j*_ equals to 1 if *p*_*j*_ belongs to *c*_*j*_ or 0 otherwise. This work sets *k* = 2 for atrial and ventricular. After *k*-means, the two cluster centers are connected to form a line segment. The perpendicular bisector of the line segment cuts the heart mask into two halves. The final mask of this step is the first half as shown in Fig. 3d. Depending on recording angles, the final half-heart mask could be either an atrial or ventricular.

### Heartbeat signal extraction

In this step, the brightness intensity of each video frame inside the half-heart mask is extracted to form an h-signal. For the h-signal of a video, the *y*-value at position *x* is defined as the brightness intensity sum of all pixels in the *x*-th frame of that video. Peaks in an h-signal correspond to heartbeats (Fig. 1). Before peak detection, two filters are introduced in this step to enhance signals. The first filter uses moving average to reduce high frequency noise; the second filter uses the Empirical Mode Decomposition (EMD) method [28] to reduce low frequency noise.

In an h-signal, the variation of brightness intensity comes from the distribution change of blood cells. The distribution of blood cells has a repetitive pattern related to heartbeats but not stable. Therefore, h-signals are usually jagged. A jagged signal could be modelled as a target signal (i.e. heartbeat in this work) mixed with noise signals of frequency higher than the target signal [29]. Moving average is a widely adopted technique to reduce high frequency noise. In this work, the exponential moving average (EMA) is adopted as a low-pass filter. The EMA method is model-free and has been widely used in time series analyses [30]. In EMA, recent data points have higher weights than older ones [31]. The smoothed (EMA-transformed) h-signal is the output of this filter.

**Fig. 5:**
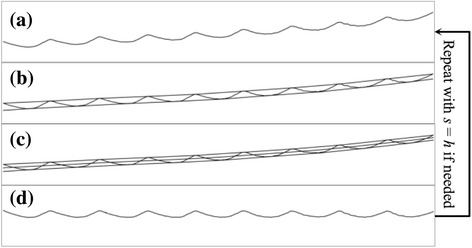
Workflow of the Empirical Mode Decomposition-based filter: (**a**) input signal, *s*; (**b**) upper and lower envelopes; (**c**) mean curve, *m*; (**d**) IMF component, *h* = *s* - *m*

In addition to high frequency noise, the smoothed h-signals of some zebrafish larva videos have low frequency noise such as the light curve in Fig. 1b. Visually, smoothed h-signals with low frequency noise have an overall increasing/decreasing trend. The overall trend could be modelled as adding a signal of frequency much lower than the target signal. To deal with this problem, this work proposes a high-pass filter based on EMD. The EMD method is a way to decompose an input signal into intrinsic mode functions (IMFs), which represent a collection of simple oscillatory modes that compose the input signal. In an IMF, by definition, the number of extrema is equal to or at most differ by one with the number of zero-crossings [28]. Figure 5 shows the workflow of the EMD-based filter. First, the smoothed h-signal is used as the initial signal, *s*. Second, the local maxima of *s* are connected as an upper envelope; while the local minima of *s* are connected as a lower envelope. Third, the mean curve, *m*, between the upper and lower envelopes is interpolated. Fourth, the component, *h*, is calculated as the difference between *s* and *m*. If *h* is not an IMF, the above four operations are repeated with *s* = *h* to obtain a new component. The entire loop stops when some component fulfills the requirements of IMF. The final component is the output of the high-pass filter as well as the output of the h-signal extraction step. The subsection “Effects of half-heart mask generation” discusses the low frequency noise and the effects of the proposed EMD-based filter on it.

### Heart rate calculation

This step calculates heart rates based on peaks in h-signals. Common recording operations (e.g. setup and remove the camera) might introduce fluctuations at the start and end of a video. To preclude such interferences, only the middle 20% time of an h-signal is considered for heart rate calculation. If the duration of an h-signal is less than 15 s, the middle 3 s are considered. In this step, the input h-signal is decomposed into data points. Each data point is the brightness intensity sum of a video frame. A data point is detected as a peak if its value is greater than its two consecutive points. Suppose *n* peaks are detected in the considered time span, *t*. The conventional formula of heart rate is *n*/*t*. This formula assumes that the time span contains roughly *n* periods. In practice, however, the time from the span start to the first peak plus the time from the last peak to the span end is not guaranteed to be a complete period. To prevent such an uncertainty, this work proposed a heart rate formula that trims the time outside the first and last peaks:

1 $$ \frac{n-1}{t_n-{t}_1} $$

where *t*_*n*_ and *t*_1_ are the timestamps of the last and first peaks, respectively, in the considered time span.

## Results

The subsection “Evaluation indices” introduces four evaluation indices. The subsection “Performance of heartbeat rate calculation” presents a validation that compared the heart rates calculated by the proposed method against those by manual counting. The subsection “Effects of half-heart mask generation” explores the effect of the proposed half-heart mask. The subsection “Larva sliding problem and the EMD-based filter” discusses a larva sliding problem and the proposed EMD-based filter.

### Evaluation indices

Table 1 shows the four error indices adopted in this work to evaluate heart rate calculation. The first index is mean absolute error (MAE), which is the expected difference of each estimation to the answer. The second index is root-mean-square error (RMSE), which squares each error and then reports the square root of the average of the squared errors. This square-then-root operation amplifies extreme values, which makes RMSE an indicator of outliers. The third and fourth indices are relative MAE (rMAE) and RMSE (rRMSE), which transform each error to its relative form (i.e. divide the error by the answer) before other calculations.

**Table 1:** Evaluation indices

Index	Abbreviation	Equation^a^
Mean absolute error	MAE	$\frac{1}{n}\sum \left|{e}_i-{a}_i\right|$
Root-mean-square error	RMSE	${\left(\frac{1}{n}\sum {\left|{e}_i-{a}_i\right|}^2\right)}^{1/2}$
Relative MAE	rMAE	$\frac{1}{n}\bullet \sum \left|\frac{e_i-{a}_i}{a_i}\right|$
Relative RMSE	rRMSE	${\left(\frac{1}{n}\sum {\left|\frac{e_i-{a}_i}{a_i}\right|}^2\right)}^{1/2}$

^a^The definition of the abbreviations used: *n* is the number of samples, *e*_*i*_ is the estimation for the *i*-th sample, and *a*_*i*_ is the answer of the *i*-th sample. For heartbeat detection, *e*_*i*_ and *a*_*i*_ are the number of heartbeats of the *i*-th video. For heart rate estimation, *e*_*i*_ and *a*_*i*_ are the heart rates of the *i*-th video

### Performance of heartbeat rate calculation

The proposed method was applied to calculate the heartbeat rates of 32 zebrafish larval cardiac videos. Each video was also counted by 11 operators for manual counting results. The 11 operators came from a bioinformatics laboratory with basic training. In nature, the heart rates delivered by an operator have certain differences when counting the same video multiple times. To alleviate such manual instability, a video was counted 30 times by an operator. With 30 repeated counting results of 11 operators, this work defined two kinds of manual errors: (a) individual error, which measures the variation between repeated experiments of an operator (i.e. human instability) and (b) crowd error, which measures the variation between different operators. The answer of a video for computational results and for crowd error was the average of all manual counting results for that video. The answer of a video for individual error was different by operator, where the answer of an operator was the average of his 30 counting results.

Table 2 shows the error of the proposed method and of manual counting in heartbeat detection and heart rate calculation. The performance of this work with the main features (the half-heart mask and noise filters described in the subsections of “Half-heart mask generation” and “Heartbeat signal extraction”) disabled, denoted as “This work (-)” in Table 2, is also provided. For heartbeat detection, the average error was less than one (MAE = 0.844), indicates that the proposed method is accurate. However, the manual counting achieved better performance in terms of MAE, RMSE and rMAE. The relatively bad rRMSE of manual counting was caused by a few outliers—operators whose errors were extremely large than those of others. This reveals a problem of manual counting—the operator must be carefully selected. This problem is a critical advantage of automatic counting. Finally, the MAE, RMSE, rMAE and rRMSE of this work were 50.9%, 63.7%, 52.7% and 65.7% to those of this work (−). This gap reveals the importance of the proposed half-heart mask and noise filters, which are further analyzed in the next two subsections.

**Table 2:** Performance of heart rate calculation

Method	MAE	RMSE	rMAE	rRMSE
Number of heartbeats				
This work	0.844	1.490	7.0%	12.4%
Individual^1^	0.498	0.673	3.3%	17.3%
Crowd^2^	0.524	0.848	3.5%	21.9%
This work (−)^3^	1.656	2.339	13.3%	18.8%
Heart rate				
This work	0.054	0.071	1.8%	2.4%
Individual	0.073	0.106	2.1%	5.6%
Crowd	0.089	0.167	2.5%	8.8%
This work (−)	0.338	0.528	11.1%	17.4%

The best performance of each evaluation index in heartbeat detection and in heart rate calculation are highlighted in bold. ^1^Error of manual counting, where the average of an operator was used as the answer for his 30 results. ^2^Error of manual counting, where the average of all operators was used as the answer. ^3^Stands for “This work minus,” in which main features (the half-heart mask and noise filters) of this work were disabled

For heart rate calculation, this work achieved the best performance in all evaluation indices. Note that the error of number of heartbeats and that of heart rate cannot be compared directly since they are in difference scales. The time span of consideration (the denominator of Eq. 1) for the 32 videos ranges from 2.3 to 6.3. Namely, an undetected heartbeat, which contributes 1 absolute error in terms of heartbeat, contributes about 0.16 to 0.43 absolute error in terms of heart rate. Although the number of heartbeats (*n* in Eq. 1) was less accurate than manual counting, it was compensated by the duration (the denominator of Eq. 1). The instability of counting timing on the first (*t*_1_ in Eq. 1) and last (*t*_*n*_ in Eq. 1) cycles harmed the performance of manual counting. Fig. 1 illustrates a typical manual error in heart rate calculation—the counting timings (vertical dotted lines) did not locate at the h-signal peaks. When watching a video, an operator naturally figures out a visual pattern to recognize a heartbeat. This visual pattern is repetitive (i.e. the relative location of the manual counting timing in each h-signal cycle is similar as those in other cycles) but varies among individuals (i.e. the relative locations of the manual counting timing in an h-signal are different by operator). In addition to the crowd error of different visual patterns, there is always individual error. It is hard for human to count at exactly the same timings when counting the same video multiple times. The displacement of the first (*t*_1_) and the last (*t*_*n*_) counts leads to individual error. This work outperformed manual counting in heart rate calculation because of eliminating such instability. Finally, as in heartbeat detection, the half-heart mask and noise filters played important roles in heart rate calculation. The MAE, RMSE, rMAE and rRMSE of this work were 15.8%, 13.4%, 16.4% and 14.0% to those of this work (−). The gap was larger than that in heartbeat detection.

In summary, heart rates calculated by the proposed method and by manual counting were accurate (MAE < 0.1 and rMAE < 5%). The performance of manual counting depended on the operator (relatively large RMSE and rRMSE). The proposed half-mask mask and noise filters largely reduced the heart rate error (reduced more than 80% error in all evaluation indices). These results indicate that the proposed method can accurately and stably calculate heart rate from zebrafish larval cardiac videos.

### Effects of half-heart mask generation

As described in the subsection “Half-heart mask generation”, the automatic half-heart mask generation of this work has four filters. The first two filters ask the brightness intensity of the generated region to be highly varied. The third filter asks the region to be big and be circle-like. The fourth filter asks the brightness intensities of pixels in the region to vary in a similar phase. This subsection discusses the effects of these filters on heart rate calculation. The heartbeat error is omitted here since it is alleviated by the trimmed version of heart rate calculation and is not critical to the proposed method.

**Fig. 6:**
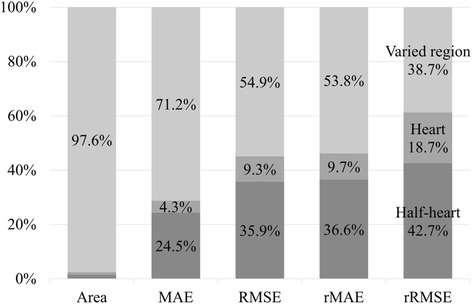
Contribution of each mask generation step. For each bar, the steps from top to bottom are varied region, heart region and half-heart region. For the Area bar, the contributions of heart and half-heart regions are 0.8% and 1.6%, respectively

Table 3 shows the heart rate errors of the proposed method with the four filters enabled incrementally. The performance of video mask does not match that of this work (−) in Table 2 because the noise filters (subsection “Heartbeat signal extraction”) were enabled. The varied region mask filtered out more than 95% of the area. The area of the final half-heart mask was only 1.4% to the entire video, which is close to the smallest heart-to-video ratio of the testing videos. All evaluation indices show a strictly decreasing trend as the filters enabled incrementally. Namely, these filters successfully identified sub-regions that are more critical than the regions delivered by the previous filters. Fig. 6 compares the performance improvement among filters. In general, the varied region mask contributed the largest improvement. The half-heart mask had the second largest contribution and the heart mask had the least contribution. The contribution of the heart mask was small because it also amplified the double-peak issue—the brightness intensity in the entire heart region has two peaks in a heartbeat cycle—while filtering out region of non-interest. Therefore, some improvement was neutralized. In this regard, the half-heart mask then recovered the improvement of focusing on heart region by solving the double-peak issue. Moreover, the contributions of the half-heart mask in RMSE and rRMSE were relatively larger than those in MAE and rMAE. The contribution of the half-heart mask exceeded that of the varied region mask in rRMSE (42.4% vs. 38.2%). These results suggest that the half-heart mask played a relatively critical role in reducing outliers and in the stability of the proposed method.

**Table 3:** Effects of the half-heart mask generation on heart rate estimation

Mask	Area^a^	MAE	RMSE	rMAE	rRMSE
Video^b^	787.2 k	0.491	0.665	11.1%	17.4%
Varied region^c^	30.2 k	0.180	0.339	6.1%	11.6%
Heart^d^	23.7 k	0.161	0.284	5.2%	8.8%
Half-heart^e^	11.3 k	0.054	0.071	1.8%	2.4%

^a^Area of the mask (unit: pixel). Heartbeat signals were extracted from the ^b^entire video (i.e. no filter of the half-heart mask generation was enabled), ^c^highly varied region (the first two filters were enabled), ^d^heart region (the first three filters were enabled) and ^e^half-heart region (all filters were enabled)

In summary, the large contribution of the varied region mask came from filtering out a large area. The heart mask yielded a relatively small contribution, but it is a necessary step for the half-heart mask. The large contribution of the half-heart mask came from accurately identifying pixels whose brightness intensities vary in a similar phase. This phase consistency was important to enhance stability.

### Larva sliding problem and the EMD-based filter

**Fig. 7:**
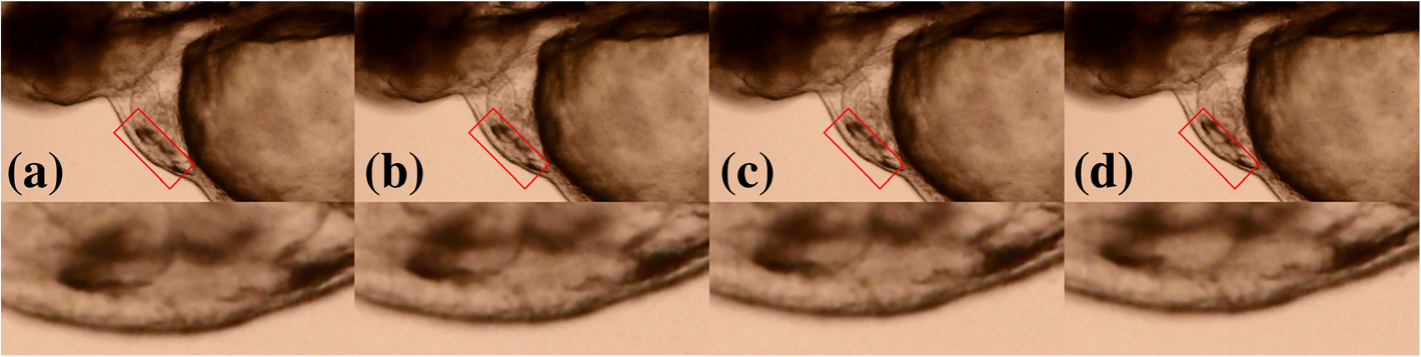
A zebrafish video with larva sliding problem (ID: MVI_5269). The timestamps of (**a**) to (**d**) are 0, 7, 13 and 20 s. The top area shows the full video frame and the bottom area zooms into the red rectangle. The bottom area shows that the larva in the video slides upward. Namely, the larva slides towards the top right corner of the full video frame. In a video frame, the larva is darker than the background. If a larva slides into the screen when recording, the extracted h-signal has an overall decreasing trend; conversely, if a larva slides out the screen like this sample video, the extracted h-signal has an overall increasing trend as shown in Fig. 1b

**Fig. 8:**
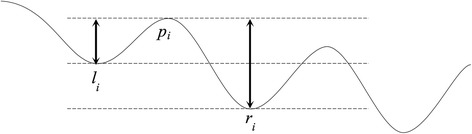
A schematic diagram of the proposed tp-index

Although the zebrafish larvae in this work were mounted on slides, the sliding of larva was still observed in some videos. Fig. 7 shows a sample video with the larva sliding problem, which leads to a global “tilting” preference of h-signals. The EMD-based filter of this work was developed to solve the tilting preference by removing the low frequency noise in h-signals. To evaluate the effect of the EMD-based filter, this work proposed an index to measure the tilting preference (denoted as tp-index):

$$ 1-\frac{1}{n}\sum \limits_{i=1}^n\frac{\min \left({p}_i-{l}_i,{p}_i-{r}_i\right)}{\max \left({p}_i-{l}_i,{p}_i-{r}_i\right)} $$

where *n* is the number of peaks, *p*_*i*_ is the *i*-th peak, *l*_*i*_ is the left trough of *p*_*i*_, while *r*_*i*_ is the right trough of *p*_*i*_ (Fig. 8). The sub-equation within the summation measures the ratio of the smaller peak-trough difference of the *i*-th peak to the larger one. The ratio decreases when the titling preference increases. The tp-index is normalized to a range of [0, 1], from small to large tilting preference. Videos with large tp-indices were manually reviewed and confirmed to have larva sliding.

With the tp-index, the 32 testing videos were divided into two groups. The first group, denoted as large-tp group, included ten videos with tp-index > 0.5; the other group, denoted as small-tp group, included 22 videos with tp-index < 0.5. The EMD-based filter was expected to have a larger effect on the first group than that on the second group. Table 4 shows the performance on the two groups with and without the EMD-based filter. The tp-index difference between two groups was large (0.623 vs. 0.300). The tp-index average of the large-tp group exceeded 0.5, indicating the ten videos suffered from serious larva sliding conditions. The EMD-based filter largely decreased the tp-index of this group (0.623–0.256 = 0.367). A smaller improvement (0.300–0.120 = 0.180) was also observed in the small-tp group. With reducing the tilting preference, the EMD-based filter improved all evaluation indices on both groups. The performance of EMD (+) in the large-tp group was close to those of EMD (−) and EMD (+) in the small-tp group. This indicates that the EMD-based filter successfully solved the larva sliding problem.

**Table 4:** Effects of the EMD-based filter

	tp-index	MAE	RMSE	rMAE	rRMSE
Large tilting preference (10 videos)					
EMD (−)^1^	0.623	0.746	0.925	26.4%	32.5%
EMD (+)^2^	0.256	0.062	0.095	2.2%	3.3%
Small tilting preference (22 videos)					
EMD (−)	0.300	0.065	0.090	2.2%	2.9%
EMD (+)	0.120	0.049	0.057	1.7%	1.9%

Heartbeat signals were extracted ^1^without and ^2^with the proposed EMD-based filter

## Discussion

The proposed method has been tested on two videos provided in [10], where one is a fly heart (BTN4602-RR-Ocorr-Sup_85747a.mov) and the other is a mouse heart (BTN4602-RR-Ocorr-Sup_85750a.mov). The program output of the two videos can be found in our online demo page (https://merry.ee.ncku.edu.tw/zebrafish/). Table 5 shows the results.

**Table 5:** performance on two videos of other species

	Fly heart		Mouse heart	
Method	# of heartbeats	Heart rate	# of heartbeats	Heart rate
Manual counting	61	2.01	12	1.278
This work	61	2.04	12	1.667

Although making a conclusion based on only two independent videos might be too arbitrary, the results reveal the potential of the proposed method on heart videos of other species.

## Conclusions

This work introduced a pipeline that calculate heart rate from zebrafish larval cardiac videos. The proposed method contains filters to identify the heart region automatically and can be performed without pre-selecting a region of heart manually. It also contains filters to withstand background fluctuations during the video recording stage, which makes recorders generate usable videos easily. The experimental results show that the proposed method does not lose accuracy while largely reducing the labor cost in long-period heartbeat counting. The automatic and accurate heart rate calculation method proposed in this work can be extended to calculate other cardiac functions like heartbeat regularity.

### Online demonstration

As the target of the proposed method is video, an online demo page at https://merry.ee.ncku.edu.tw/zebrafish/ was setup to supplement the static figures in this manuscript. The source code of the proposed method is deposited in Github at https://github.com/mbilab/zebrafish.

## Additional file

Additional file 1: **Table S1.** Performance of heart rate estimation with different color-to-grayscale methods. (DOCX 14 kb)

## Abbreviations

CHD: Congenital heart disease
dpf: Days post fertilization
EMA: Exponential moving average
EMD: Empirical mode decomposition
h-signal: Heartbeat signal
IMFs: Intrinsic mode functions
MAE: Mean absolute error
rMAE: Relative MAE
RMSE: Root-mean-square error
ROI: Regions of interest
rRMSE: Relative RMSE
this work (−): Main features (the half-heart mask and noise filters) of this work was disabled.
tp-index: Index to measure the tilting preference
