# Supplementary material for: An automatic method to calculate heart rate from zebrafish larval cardiac videos
Source: BMC Bioinformatics. 2018 May 9;19:169. doi: 10.1186/s12859-018-2166-6 (PMC5944013; doi:10.1186/s12859-018-2166-6)
Supplement: Supplementary file 1 — Table S1. Performance of heart rate estimation with different color-to-grayscale methods. (DOCX 14 kb) [file 12859_2018_2166_MOESM1_ESM.docx]

Table S1 Performance of heart rate estimation with different color-to-grayscale methods.

| Color-to-grayscale methods | MAE | RMSE | rMAE | rRMSE |
| --- | --- | --- | --- | --- |
| *G_Intensity_* | 0.083 | 0.172 | 2.9% | 6.1% |
| *G_Intensity_* with gamma correction (*G_Gleam_*) | 0.193 | 0.354 | 6.6% | 12.0% |
| *G_Luminance_* | 0.133 | 0.309 | 4.7% | 10.8% |
| *G_Luminace_* with gamma correction | 0.125 | 0.276 | 4.2% | 9.2% |
| *G_Luma_* | 0.199 | 0.352 | 6.6% | 11.6% |
| *G_Luma_* without gamma correction | 0.054 | 0.071 | 1.8% | 2.4% |
| *G_Lightness_* | 0.180 | 0.312 | 6.5% | 11.3% |
| *G_Lightness_* with gamma correction | 0.120 | 0.200 | 4.2% | 6.8% |
| *G_Value_* | 0.142 | 0.260 | 5.0% | 9.2% |
| *G_Value_* with gamma correction | 0.156 | 0.261 | 5.6% | 9.4% |
| *G_Luster_* | 0.125 | 0.291 | 4.4% | 10.1% |
| *G_Luster_* with gamma correction | 0.199 | 0.366 | 7.1% | 13.0% |

The MAE, RMSE, rMAE and rRMSE stand for mean absolute error, root-mean-square error, relative MAE and relative RMSE, respectively. The detailed definition of the four metrics can be found in the “Evaluation indices” subsection of the main manuscript. The details the of 12 color-to-grayscale methods can be found in [1].

1. Kanan C, Cottrell GW: **Color-to-grayscale: does the method matter in image recognition?** *PloS one* 2012, **7**(1):e29740.
